# With a new clip technique surgically inducing varicocele in Sprague-Dawley rats

**DOI:** 10.1186/s12894-018-0350-7

**Published:** 2018-06-07

**Authors:** Wen-bin Guo, Cheng Yang, Jun Bian, Hui Xia, Jian-kun Yang, Qi-zhao Zhou, Ming-kun Chen, Kang-yi Xue, Wan-song Zhang, Peng Wang, Xin Li, Cun-dong Liu

**Affiliations:** 1 Department of Urology, the Third Affiliated Hospital of Southern Medical University, No. 183 West Zhongshan Road, Tianhe District, 510630 Guangzhou, People’s Republic of China; 2 Center for Clinical Research and Innovation, Shenzhen Hospital of Southern Medical University, Shenzhen, People’s Republic of China

**Keywords:** Clip, Varicocele, Spermatic vein, Rat model

## Abstract

**Background:**

We introduced and recreated a more consistent and effective experimental varicocele rat model by a new clip technique.

**Methods:**

A total of 40 rats were numbered and randomly assigned to 5 groups of 8 each, including sham surgery (Group I), conventional (Group II) and clip groups with 0.7, 0.8, 0.9 mm gap widths, respectively (Group III, IV, V). All of the rats in each group were sacrificed at 8 weeks after initial surgery, and the rats forming out with less than 1 mm diameter of left spermatic vein or no presence of the pampiniform plexus dilation were excluded from the experimental groups. The left spermatic vein (LSV) diameter, testicular weight, left kidney weight to body weight coefficients, kidney and testicular histology were determined.

**Results:**

The baseline mean diameter of the LSV in Group I, II and III was 0.22 ± 0.02, 0.23 ± 0.02 and 0.22 ± 0.03 mm, respectively (*P* = 0.7504). At 8 weeks after initial surgery, varicocele was successfully created in 6/8 (75%), 7/8 (87.5%), 3/8 (37.5%), 3/8 (37.5%) in GroupII-V, no varicocele was observed in Group I. In Group I, II and III, no pathological changes were observed and the left kidney weight to body weight coefficients showed no significant differences. The diameter of LSV was remarkably increased both in Group II and III compared to Group I (1.72 ± 0.13, 1.57 ± 0.19 and 0.25 ± 0.02, respectively), and Group II and III had a smaller testicular weight than the rats in Group I (1.67 ± 0.05, 1.62 ± 0.06, and 1.92 ± 0.12, respectively).

**Conclusions:**

With a new clip technique, surgically inducing varicocele rat model becomes convenient and safe. This appears to improve the effectiveness of the model and this innovation may allow us to further understand the pathophysiology of varicocele.

## Background

Varicocele (VC) is a condition characterized by the spermatic varicosity or abnormal dilation of testicular pampiniform venous plexus, and is present in 35% of men with primary infertility and in 70 to 81% with secondary infertility [1, 2]. Although it has been considered to be associated with male infertility, the exact pathophysiology of the varicocele is incompletely understood [3].

Specific questions regarding the pathophysiological of varicocele are even difficult to address in human. Several factors are often taken into account, such as limitedly acquisition of tissue, forbidden invasive experiment, or other indefinite characteristics [4]. Thus, this highlights the key role in the use of animal models. To date, the most widely used experimental varicocele rat model is to investigate the mechanisms underlying the varicocele-related male infertility. However, experimental rat model induced by the conventional technique has varied success [5–7]. In the present study, for the first time we used a clip technique mimicking the nutcracker phenomenon suffering a partial occlusion of the renal vein combined with ligation of the branches of the LSV to develop experimental rat varicocele model, which was more consistent and effective to help to create varicocele rat model.

## Methods

### Animal care

This study was approved by the animal care and ethics committee of the Southern Medical University. A total of 40 adult male specific-pathogen free (SPF) Sprague Dawley rats (*Rattus norvegicus*, age range from 7 weeks to 8 weeks, weighing 250–300 g) obtained from the animal center of Southern Medical University (certificate number: SYXK [Yue] 2011–0074), were housed in a climate controlled environment with free access to food and water under a 12-h day/night cycle. A 1-week acclimatization period was allowed prior to the experimental procedures.

### Experimental design

The 40 rats were numbered and randomly assigned to 5 groups of 8 each, with the method of random digits table, including sham surgery (Group I), conventional group (Group II), clip groups (0.7 mm, 0.8 mm and 0.9 mm gap widths corresponding to Group III, IV, V, respectively) (obtained from ALCOTT BIOTECH, Shanghai, China). A sample size of 8 rats per group was sufficient according to our preliminary experiment and the previous study that the success rate of rat varicocele was ranged from 85 to 100% [8, 9]. At 8 weeks after initial surgery, all of the rats in each group were sacrificed. The postoperative outcomes were determined, including the left kidney histology, left kidney weight to body weight coefficients, the diameter of left spermatic vein (LSV), left testicular weight, histology and complications. The micrometer was used to measure the diameter of the LSV at the level of the crossing iliolumbar vein. All the operations were carried out by two skillful researchers, but they were not involved in the collection of data, analysis of results and evaluation of outcomes. All measurements were reviewed and based on agreement by 2 investigators independently. Other investigators and outcome assessors were blind to the groups.

### Experimental procedures

In the present study, we created varicocele model in male Sprague-Dawley rats according to the procedures previously described by Turner TT [4], with partially occluding left renal vein and completely ligating the branches of the LSV. All experimental procedures were performed in a sterile environment. Briefly, after 12 h fasting, the rats were anesthetized using an intraperitoneal injection of 30 mg/kg sodium pentobarbital. The experimental rats were considered anaesthetized when holding and pressing the paw did not elicit a response from the rats. A midline laparotomy incision was made from xyphoid to pubis to visualize the left kidney, left adrenal vein, the left renal vein and the LSV and its collaterals. A tunnel was made around the left renal vein by careful blunt dissection, which was cleared of adhering tissue in a position medial to the insertion of the LSV and left adrenal vein. In Group II, a 4–0 silk suture was used for partially occluding of the left renal vein around a metal wire of 0.85 mm diameter at the point. The wire was then removed from the ligature, allowing the vein expand to an external diameter. In Group I, the rats with sham operation underwent a similar procedure that the left renal vein was dissected free but not ligated. In Group III, IV, and V, the clips with 0.7 mm, 0.8 mm and 0.9 mm gap widthsinstead of suture ligature was placed at the same point on the renal vein. Finally, the collaterals of the LSV were completely occluded and then the incision was closed in two layers with 3–0 silk sutures.

### Histopathology assessment

After 8 weeks, the testes and kidneys were harvested and weighted. Half of each kidney and testis was fixed in 4% paraformaldehyde and modified Davidson’s Fluid’s solution [10], respectively. These were postfixed in 70% alcohol and embedded in paraffin blocks after tissue processing. Sections (5 μm) from testes were obtained, deparaffinized and stained with hematoxylin and eosin. Disintegration of the germinal epithelium and degenerative appearance in each group was observed by light microscopy. For kidney histology, sections were stained with periodic acid-Schiff (PAS) and Masson’s trichrome (Baso, Zhuhai, China). Histopathology analysis was performed by two uropathologists independently.

### Statistical analysis

Statistical analysis was performed using SPSS 20, and all data are presented as the mean ± SD. Comparisons of all 3 groups and 2 groups were made using the Kruskal-Wallis and the Games-Howell test for continuous variables, respectively. A value of *P* < 0.05 was considered statistically significance.

## Results

### The characteristics of experimental varicocele development in each group

**Fig. 1:**
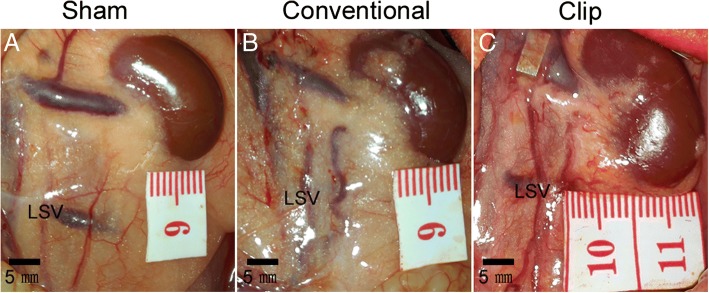
The left spermatic vein (LSV) varicosity. At 8 weeks after initial surgery, (**a**, **b**, **c**) the LSV showed obvious dilatation in the conventional and clip groups compared to sham surgery. Scale bar = 5 mm

Some complications occurred in study. For example, Group II had 1 rat tearing of vein or bleeding and 1 rat pyonephrosis, which lead to model failure. In Group IV and V, there were 3/8, and 5/8 clips showing slipping off the renal vein, respectively, while in Group III no severe complication happened and all rats had successful except for one rat failing to form varicocele. According to the criteria of VC model, the rats forming out with more than 1 mm diameter of left spermatic vein or presence of the pampiniform plexus dilation [4] in each group were included in the experimental groups [Fig. 1]. According to Table 1, we selected Group III for further analysis, excluding Groups IV and V because that the number of inducing varicocele is not enough to allow for extrapolations from the proposed statistics.

**Table 1:** The characteristics of experimental varicocele development in each group

Group	Number	Survival rate	Complications	Varicocele	Success rate
I^a^	8	100%	0	0	0
II^a^	8	100%	3^b^	6	75%
III^a^	8	100%	0	7	87.5%
IV^a^	8	100%	0	3	37.5%
V^a^	8	100%	0	3	37.5%

^a^I: Sham II: Conventional III: 0.7 mm clip IV: 0.8 mm clip V: 0.9 mm clip

^b^2 rats bleeding and 1 pyonephrosis

### Baseline anatomy and outcomes

**Fig. 2:**
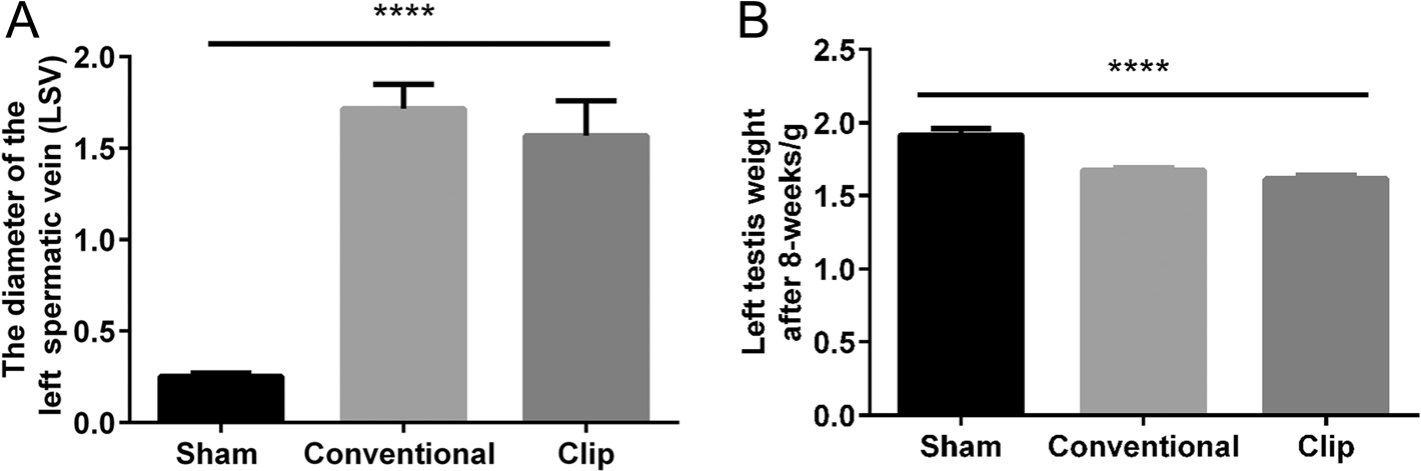
The diameter of the left spermatic vein (LSV) **a** At 8 weeks after initial surgery, the diameter of LSV was remarkably larger and **b** the left testis weight was smaller in the conventional and clip groups when compared with sham surgery (****P < 0.0001)

The mean baseline of total body weight of rats among Group I, II and III was 331.25 ± 8.27, 329.13 ± 13.66 and 326.75 ± 12.49 g, respectively (*P* = 0.6992). Baseline mean diameter of the left spermatic vein in these three groups was 0.22 ± 0.02, 0.23 ± 0.02 and 0.22 ± 0.03 mm, respectively (*P* = 0.7504). At 8 weeks after initial surgery, the size was remarkably increased when measuring the diameter of LSV both in Group II and III compared with Group I (1.72 ± 0.13, 1.57 ± 0.19 and 0.25 ± 0.02, respectively, *P* < 0.0001, Fig. 2a). No significant difference was observed between Group II and III (*P* = 0.275). Group II and III had smaller testicular weight compared with Group I (1.67 ± 0.05,1.62 ± 0.06, and 1.92 ± 0.12 respectively, *P* < 0.0001, Fig. 2b). There was also no difference between Group II and III (*P* = 0.196).

### Evaluation of experimental varicocele model

**Fig. 3:**
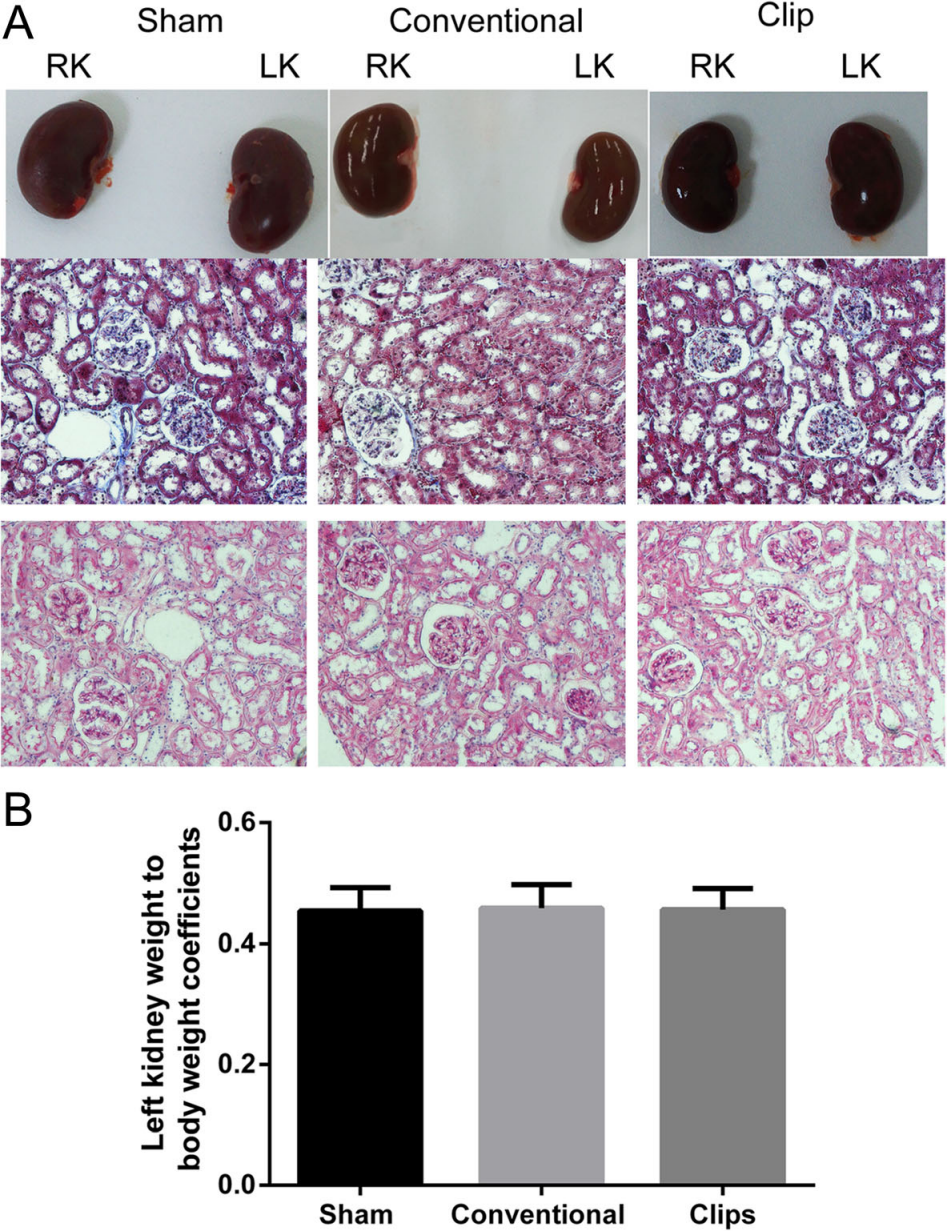
Histopathology assessment of left kidney. (**a**) (a, b, c) Masson’s Trichrome staining and (d, e, f) PAS staining of the left kidney showed no significantly pathological changes in the sham, conventional and clip groups after 8 weeks. Scale bar = 50 μm. Right kidney: RK, Left kidney: LK. (**b**) The left kidney weight to body weight coefficients showed no significant differences among these three groups

**Fig. 4:**
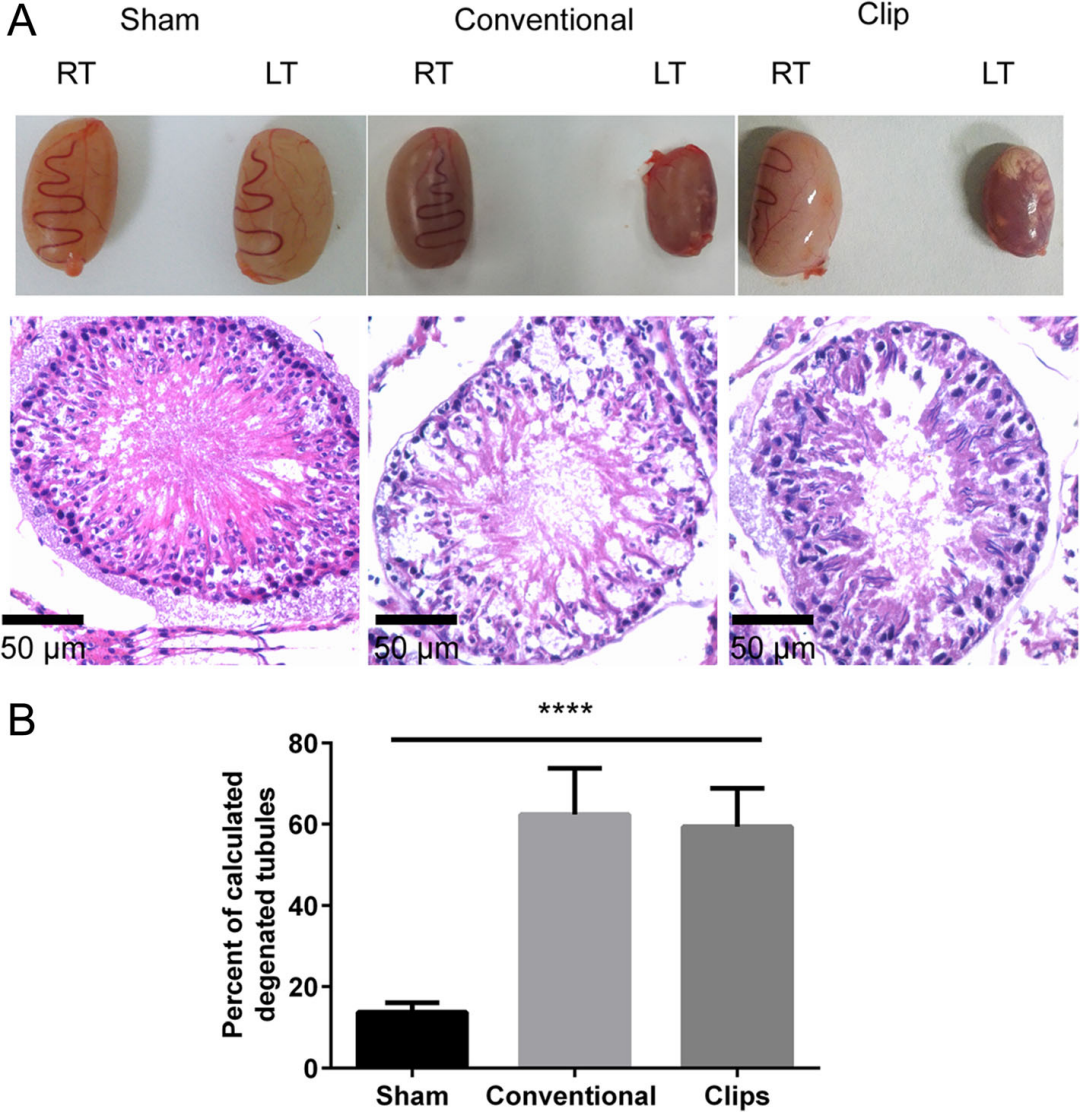
Hematoxylin and eosin stained rat left testicular tissues after 8 weeks. **a** When compared with the sham group, rats showed an apparent degeneration of the tubules (magnification 40X) and **b** the percentage of degenerated tubules was significantly higher (****P < 0.0001) in the conventional and clip groups. Scale bar = 50 μm. Right testis: RT, Left testis: LT

After 8 weeks, macroscopic changes in the left kidney were carefully identified and compared with the right kidney. No pathological changes were observed in Group I, II and III [Fig. 3a]. The left kidney weight to body weight coefficients showed no significant differences among these three groups [Fig. 3b]. When calculating the numbers of degenerating tubules and compared the degenerating tubule percentages among these three groups, we found the percentage both in Group II and III were statistically significantly increased as compared with Group I [Fig. 4], which indicate impairment of spermatogenesis.

## Discussions

Despite the fact that varicocele associated with male subfertility is widely accepted [11], the pathophysiology of varicocele has been poorly understood. With rare exception, varicocele does occur exclusively in humans, which are more difficult to address. Thus, the use of animal models has played an essential role in understanding this condition. In 1981, Saypol et al. [12] reported that surgically induced varicocele using partial left renal vein occlusion had influence on testicular blood flow, temperature, and histology in adult rats and dogs. Subsequently, many studies investigating on the pathophysiology underlying varicocele associated with infertility were conducted by this method [4, 13–15]. As similarities existed in venous anatomy between the rat and human when left varicocele occurs [16], since then experimental varicocele in rat has been the most widely used animal model. However, it has resulted in varied success except for interlaboratory and interoperator intrinsic variability [6, 7].

Previous studies on the variable vascular anatomy in rats may contribute to develop varicocele rat model [5, 17, 18]. In most rats, the left venous pampiniform plexus drains primarily into the left common iliac and a smaller internal spermatic vein to the left renal vein [5, 19]. In the present study, we created varicocele rat model by partially occluded the renal vein combined with completely ligation of the collaterals of the left spermatic vein with minor modifications. Furthermore, to improve the efficacy and create a consistent rat varicocele model, we used a novel clip technique usually applied to produce hypertension in the 2K1C model (2-kidney, 1-clip) [20]. The clip has more advantages of accurate and consistent reduction of renal vein diameter than suture ligation. To date, a key factor in current varicocele model is the potential for a correct and consistent degree of obstruction as a result of too much reduction in renal vein resulting necrosis or too little construction failing to form varicocele. Thus, clips with 0.7, 0.8 and 0.9 mm gap widths were selected in our study according to the 0.85 mm diameter as criteria reported by Turner TT [4]. At 8 weeks after initial operation, clip with 0.7 mm gap widths was appropriate and selected for further analysis in the study. No macroscopic changes and histological findings in the kidneys were observed among Group I, II and III, indicating there was no renal lesion paralleling the clinical findings of varicocele in humans [21]. The Group III had larger mean diameter of left spermatic vein and worse testicular histology than the Group I, similar to Group II but showing no significant difference. These results were accordance with these studies previously reported [18, 22].

Moreover, success rate of experimental varicocele rat model, up to a certain extent, depends on the complications during the operation [9]. In this study, the success rate in group 2 is only 75% (6/8). Many factors may account for this, including vascular injury, pyonephrosis. Sometimes when blinded to dissect behind the renal vein, inadvertent puncture or tear of the vein often occurs, resulting in excessive bleeding. Vascular injury also happens just when ligating the left renal vein. In Group II, 1 rat with pyonephrosis is mainly due to mistakenly ligation of ureter adjacent to spermatic vein, leading to obstructive hydronephrosis and infection. Using clip technique, we found no complications in Group III, and the success rate is 87.5% (7/8) except for 1 rat failing to form a varicocele because of one clip dislodging from the renal vein. Although the effects of clip technique on varicocele in this study, the convenient and safe model constitutes a basis for investigating on male infertility underlying varicocele, which is still further evaluated in subsequent studies that also need to expand the sample size.

There were also some inadequacies in this model. In Group IV and V, these clips were slipped off or dislodged from the renal vein, resulting in failing to form varicocele. The clip needs to be designed to eliminate the possibility of dislodgement after implantation with some modifications. In addition, although clip with 0.7 mm gap widths was appropriate in this model, the different diameters of clips should be further investigated in different characteristics of species, age, and weight in rat.

## Conclusion

With a new clip technique, surgically inducing varicocele rat model becomes convenient and safe. This appears to improve the effectiveness of the model and this innovation may allow us to further understand the pathophysiology of varicocele.

## Abbreviations

2K1C: 2-kidney, 1-clip
LSV: Left spermatic vein
PAS: Periodic acid-Schiff
SD: Standard deviation
SPF: Specific-pathogen free
VC: Varicocele
